# Quantitative Methods for the Detection of Tau Seeding Activity in Human Biofluids

**DOI:** 10.3389/fnins.2021.654176

**Published:** 2021-03-19

**Authors:** Aurelien Lathuiliere, Bradley T. Hyman

**Affiliations:** ^1^Alzheimer Research Unit, Department of Neurology, Massachusetts General Hospital, Charlestown, MA, United States; ^2^Harvard Medical School, Boston, MA, United States

**Keywords:** tau, seed, biomarker, Alzheimer’s disease, cerebrospinal fluid

## Abstract

The ability of tau aggregates to recruit and misfold monomeric tau and propagate across brain regions has been studied extensively and is now recognized as a critical pathological step in Alzheimer’s disease (AD) and other tauopathies. Recent evidence suggests that the detection of tau seeds in human samples may be relevant and correlate with clinical data. Here, we review the available methods for the measurement of such tau seeds, their limitations and their potential implementation for the development of the next-generation biomarkers.

## Introduction

The accumulation and deposition of tau protein aggregates in the human brain is a hallmark of Alzheimer’s disease (AD) and other tauopathies. The capacity of certain toxic tau species or conformers to propagate from one cell to another and template or “seed” endogenous tau in a prion-like mechanism has been now widely studied and demonstrated in various disease models. *In vitro*, the aggregation of recombinant tau into paired helical filaments is accelerated by the addition of pre-formed seeds, suggesting a seeded nucleation process that results in the elongation of aggregates (Friedhoff et al., 1998; von Bergen et al., 2000). In cellular models, tau aggregates are internalized and induce aggregation of intracellular monomeric tau (Frost et al., 2009; Guo and Lee, 2011; Kfoury et al., 2012). In transgenic mice overexpressing mutant tau, the injection of synthetic tau fibrils or brain lysates from patients with tauopathies induces aggregation of tau and the spread of the pathology to distant brain regions (Clavaguera et al., 2009; Iba et al., 2013). The critical role of tau seeding and spreading in the pathogenesis of AD is further supported by the stereotypical progression of tau pathology across brain regions that has been described by neuropathological studies (Braak and Braak, 1991) and confirmed more recently by molecular imaging studies (Cho et al., 2016; Scholl et al., 2016; Schwarz et al., 2016). Moreover, seed-competent tau is detected in the synaptic compartment in brain regions along the Braak staging before the appearance of the pathology (Holmes et al., 2014; DeVos et al., 2018). This soluble seed-competent species represent a small percentage of total tau that elutes as a high molecular weight (>∼300,000) fraction from a size exclusion chromatography column (Takeda et al., 2015, 2016).

Using modern AD biomarkers including tau and amyloid imaging by positron emission tomography (PET), volumetric magnetic resonance imaging (MRI), and cerebrospinal fluid (CSF) measurement of Aβ and tau (and in the near future plasma levels of Aβ and tau), one can accurately diagnose AD at a presymptomatic or prodromal stage [reviewed in Cohen et al. (2019), Zetterberg and Bendlin (2020)]. However, there is currently no available biomarker that can predict on a time scale the clinical fate of an individual with evidence of brain amyloid or tau neuropathology. Recently, we have demonstrated that the tau seeding activity in postmortem AD brain extracts is quantitively and qualitatively correlated with disease severity and rate of progression (Dujardin et al., 2020). Therefore, the quantification of tau seeds in human biofluids such as CSF could represent a potential prognostic biomarker that may greatly improve individual patient care.

Here, we review the currently available quantitative methods to measure seed-competent tau, their advantages and limitations as well as their potential development for a broader use in a biomarker pipeline.

## Cerebrospinal Fluid Biochemical Assays

The measurement of Aβ42, total tau (*t*-tau) and phospho-tau (p-tau) in the CSF by immunoassays is a core component of AD clinical criteria (Frisoni et al., 2017; Jack et al., 2018). The typical profile in AD patients is characterized by high level of *t*-tau and p-tau and reduced level of Aβ42. The sensitivity and specificity of these measures varies greatly across studies. A recent meta analysis concluded that they may be better used to rule out the diagnosis of AD because of a greater sensitivity than specificity (Ritchie et al., 2017). High CSF *t*-tau is generally considered as a measure of acute injury or ongoing neurodegeneration (Blennow et al., 1995). It is therefore not specific for AD and is found elevated in rapidly progressive dementia such as Creutzfeldt Jakob disease or in acute traumatic brain injury or stroke (Hesse et al., 2000; Ost et al., 2006; Skillback et al., 2014). Elevated CSF p-tau, is found in AD and is therefore useful to discriminate AD from other dementia such as dementia with Lewy bodies or frontotemporal dementia (Hampel et al., 2004). Interestingly, in non-AD tauopathies (or FTLD-tau), even though pathological brain aggregates consist in phosphorylated tau, inconsistent findings are reported in the literature regarding CSF levels of *t*-tau or p-tau. Discrepant studies have reported elevated *t*-tau and p-tau (Casoli et al., 2019), high *t*-tau but normal p-tau (Foiani et al., 2019) normal level of both (Goossens et al., 2018) or even decreased level of both (Wagshal et al., 2015). Those studies rely on ELISA-based quantitation which depend on antibody epitopes. While p-181 epitope is usually used for p-tau detection, the use of other phospho-epitope could potentially help discriminate tauopathies. The CSF biomarkers are useful from a diagnostic perspective in AD. However, their performance in assessing clinical progression of the disease or conversion to dementia is variable in the literature. Some studies found a correlation between elevated *t*-tau and p-tau with faster decline or higher mortality (Samgard et al., 2010; Degerman Gunnarsson et al., 2014). Other longitudinal studies found no correlation or even a relative stability of tau levels during the course of the disease (Andreasen et al., 1999; Sunderland et al., 1999; Vemuri et al., 2009; Williams et al., 2011). A recent study found a correlation between *t*-tau and p-tau levels and faster cognitive decline in ApoE-ε4 carriers only, which confirmed their limited predictive utility (Wattmo et al., 2020). Recently, the sensitivity of assays to detect p-tau in plasma has been improved. The plasma levels that are typically measured fall in a range between 1 and 10 pg/ml. Several recent studies have demonstrated that elevated plasma p-tau (p-tau181 or p-tau217) levels can discriminate AD from controls or from other neurodegenerative dementias (Janelidze et al., 2020; Palmqvist et al., 2020; Thijssen et al., 2020). Interestingly, plasma p-tau181 seemed to correlate with CSF p-tau181 and tau burden on PET imaging (Janelidze et al., 2020). These promising results need to be confirmed in larger primary care cohorts to validate the feasibility and clinical utility of this new biomarker.

## Cell-Based Assays

**FIGURE 1:**
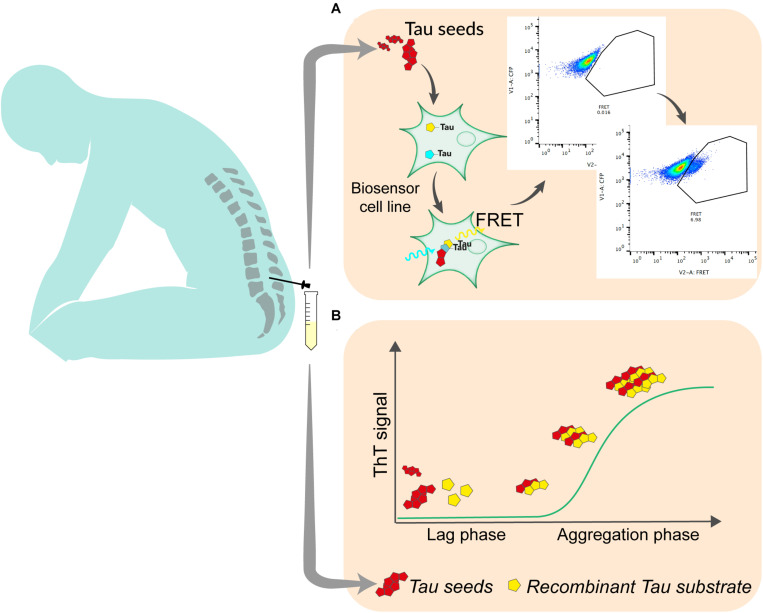
The most promising techniques for the quantitation of tau seeding activity in human biofluid. **(A)** In cell-based assay, seed-containing sample is incubated on a biosensor cell line overexpressing tau linked to either a CFP or YFP fluorescent protein. Upon aggregation, energy transfers between CFP and YFP allows for the detection of FRET signal using flow cytometry. Signal is quantified as integrated fret density which is the product between the percentage of FRET-positive cells and the median fluorescence intensity in the FRET channel. **(B)** In RT-QuIC, seed-containing material is incubated with recombinant tau substrate with thioflavin T in optimized conditions. Seed-competent material induces the aggregation of the substrate which generate ThT fluorescence that is measured over time.

The development of reporter cell lines, based on Förster resonance energy transfer (FRET) has been an important step in the understanding of the prion-like propagation of tau (Holmes et al., 2014). This type of biosensor is currently widely used to detect tau seeding activity in brain samples. It relies on the overexpression of the repeat domain (RD) of tau with the pro-aggregating P301L mutation fused to either a cyan fluorescent protein (CFP) or yellow fluorescent protein (YFP). After exposure to exogenous seed-competent tau, fluorescent reporters aggregate, which produce FRET signal that is typically quantified by flow cytometry, 24–72 h after exposure to tau seeds (Furman et al., 2015) (Figure 1). The original and most commonly used biosensor cell line is a clonal HEK293T line that was developed by the group of Marc Diamond and that is now commercially available (ATCC CRL-3275). Moreover, a similar reporter system can be used in mouse primary neuronal cells (Holmes et al., 2014). Slightly modified versions of this assay have also been tested by other groups. For instance, different fluorescent protein pairs have been used to increase the dynamic range of the assay (Chen et al., 2019). The addition of liposomes (lipofectamine) to facilitate the transduction of seeds into cells greatly increases the sensitivity of the biosensor assay but bypasses tau uptake mechanisms and therefore does not reflect seeding as it happens in the brain. A recent report suggests that the fusion of tau RD to fluorescent proteins may induce steric hindrance that avoids the elongation of tau aggregates into paired helical filaments (Kaniyappan et al., 2020b). The authors propose that the increase of FRET signal after exposure to seed-competent tau may result from cellular processes different from aggregation. Nevertheless, in a heterogeneous group of AD patients, the use of biosensor cells transduced by lipofection analyzed by live cell imaging and image processing could consistently detect a lag phase followed by an exponential elongation phase and a plateau phase in the aggregation, suggesting that the assay is relevant to seeding in the disease process (Dujardin et al., 2020). The use of such biosensor is therefore an interesting approach to quantify seed-competent tau in human biofluids. While the assay is sensitive enough to quantify seeds in postmortem ventricular CSF (Takeda et al., 2016), lumbar CSF requires concentration steps that may alter quantitative aspect of measurement (Takeda et al., 2016; Crotti et al., 2019). Thus, some optimization needs to be done to improve the assay sensitivity and tailor it for a clinical use with reasonable volume of CSF. Such optimization may include ways to enhance the aggregation of FRET probes, including using tau constructs that may be more disease-relevant such as fragments covering the whole structure of amyloid core of tau aggregates (Fitzpatrick et al., 2017), or adapting the linker between fluorescent reporters and the tau protein to avoid steric hindrance (Kaniyappan et al., 2020a). In addition, adapting the fluorescent protein pairs to increase the FRET efficiency may also increase the signal produced by reporter cell lines (Bajar et al., 2016). A cell-based assay was able to detect tau seeding in brain from various tauopathies (Sanders et al., 2014). However, whether specific probe construct will be able to discriminate seeds from different pathologies has yet to be demonstrated. Even if some adaptations need to be tested and validated, they appear realistic and may contribute to push FRET based biosensor to a clinical application. Fluorescence-based assays for routine diagnostics are already used in various clinical fields such as oncology or immunology.

## Seed Amplification Assays

The capacity of proteopathic seeds from neurodegenerative disorders to self-propagate, recruit and template the aggregation of monomers has been exploited in a wide range of biomarker assays. The demonstration that infectious prions could misfold native prion protein and generate seeds in a cell-free environment opened the way to this type of assay (Kocisko et al., 1994). Various generations of assays using either unaffected brain sample as substrate (Saborio et al., 2001) or Escherichia *coli*-produced recombinant prion protein (Atarashi et al., 2007) have been developed over the years and led to the widely used technique of Real-Time Quaking-Induced Conversion (RT-QuIC) for the diagnosis of sporadic Creutzfeldt Jakob disease (Atarashi et al., 2011; Orru et al., 2014). This system relies on the incubation in a 96-well plate, of biospecimen, typically CSF, with recombinant prion protein in excess, thioflavin T; in a defined buffer, at a controlled temperature (42°C), with shaking cycles over the course of several days. Thioflavin T fluorescence is measured every 45 min (Figure 1). The mean of highest fluorescence values over the course of the analysis is compared to control samples to determine positivity. In the presence of seeding-competent material, the typical trace starts with a lag phase that corresponds to the time needed for aggregating material to reach a concentration that can be detected by thioflavin T. The signal then reaches a plateau that reflects the conversion of all monomer substrate into amyloid (Schmitz et al., 2016). Similar methods have been used to detect seeds in synucleinopathies or tauopathies. The specificity of the seed amplification relies on the design of the substrate protein. Therefore, different RT-QuIC assays have been initially reported to detect specifically 3R, 4R, or 3R/4R seeds in corresponding tauopathies. For the detection of seeds in Pick’s disease, which is characterized by 3-repeat tau deposition, a K19 tau fragment from 244 to 372, lacking the second repeat was used (Saijo et al., 2017). The only cysteine was mutated to serine to prevent the formation of disulfide bonds during the reaction. Despite the fact that this fragment does not cover the entire structure of Pick’s disease’s tau filaments (Falcon et al., 2018), RT-QuIC could detect tau seeds in postmortem CSF with very high sensitivity (Saijo et al., 2017). A modified version of this assay, incorporating a second tau fragment covering the entire cryoEM structure of AD paired helical filaments (residues 306–378) was able to detect seeds in AD brain (Kraus et al., 2019). When an extended 3-repeat cysteine free K12 fragment spanning from 244 to 400 was used, the self-polymerization of the probe was reduced and RT-Quic assay sensitivity and specificity were consequently increased, allowing for the detection of seeds in both 3R (Pick’s disease) and 3R/4R [AD, chronic traumatic encephalopathy (CTE)] tauopathies (Metrick et al., 2020). Recently, the same group published an updated assay capable of measuring tau seeds from 4R tauopathies (progressive supranuclear palsy and corticobasal degeneration) with a sensitivity down to 2 femtograms (Saijo et al., 2020). For the first time, the authors described some signal in antemortem lumbar CSF suggesting that RT-QuIC -based assays could, with further optimization, detect, and or quantify seeds in the CSF of AD patients in the future. Interestingly, in both K12 and 4R assays the analysis of ThT amplitude can discriminate the different diseases-specific conformers (AD vs. Pick’s or PSP vs. CBD) using the same assay conditions. Combining the two assays may hence be used to infer histopathological diagnosis. One limitation of tau RT-QuIC assays is that they rely on the use of heparin to promote the templating of tau substrate. It was shown that the structure of heparin-induced tau filaments differs from those found in AD or other tauopathies (Zhang et al., 2019). It might be interesting to evaluate substrates that include some post translational modifications that have been recently associated with seeding (Wesseling et al., 2020).

## *In vivo* Seed Amplification Assays

It is now well established also that injection of tau seeds into a transgenic animal that over-expresses human tau can lead to tau aggregates after several months (Clavaguera et al., 2009, 2013; Iba et al., 2013; Ahmed et al., 2014). Interestingly, in a mouse model overexpressing equimolar amount of both 3R and 4R tau isoforms the intracerebral injection of pathological tau seeds from different tauopathies (AD, CBD, PSP, and PiD) recruited the corresponding predominant isoform (He et al., 2020). In addition, the seeds from distinct tauopathies recapitulated cell-type specificity of the pathology in the recipient animal. CBD and PSP-derived seeds induced neuronal but also oligodendrocytic and astrocytic pathology as observed in human brain. All together, these results suggest that distinct seeds may carry different conformations that lead to specific isoform recruitment and to transmission to specific cell types. Skachokova et al. (2019) extended these observations to determine if the tau present in lumbar CSF collected from AD patients might also trigger aggregation of endogenous tau, and found that, over a period of about 4 months, CSF injected intrahippocampally into young P301S overexpressing mice did indeed form aggregates, reinforcing the idea that tau seeds detected using *in vitro* assays are biologically relevant in the intact organism as well (; Dujardin et al., 2020). Although not frequently used, animal-based bioassays have been validated for clinical diagnosis (e.g., for the detection of botulinum toxin) and could potentially be used as a platform for the detection of tau seeding activity in human biofluids. However, the accuracy and feasibility of such approach still needs to be demonstrated.

## Discussion and Future Directions

The focus of biomarkers to date has been to aid in the diagnosis of neurodegenerative diseases, especially AD. This has been challenging in part because of the widespread recognition that neuropathological lesions can precede symptoms by years if not decades, so that knowing how to interpret positive results in an assay among the “controls” has been problematic. Nonetheless, largely with the aid of elegant studies in genetically defined at risk populations, PET scans for both Amyloid and tau and CSF biomarkers are well established. Yet some limitations remain: none of the tau-based markers are yet useful for non-AD tauopathies, and none of the currently available markers provide insight into the prognosis of an individual patient. Recent studies using brain tissue raise the possibility that there is considerable variability in tau post translational modifications across patients, which also is reflected in seed properties in a tau bioactivity assay (Dujardin et al., 2020; Sepulveda-Falla et al., 2020). If these alterations are also detectable in CSF, such differences may well provide insight into predicting relative rates of progression in living patients as well. Similarly, development of additional markers of synaptic structure or function, inflammatory status of glia, and blood brain barrier dysfunction may all help in providing critical information for physicians and patients. Finally, biomarkers that provide insight into rates of progression might be valuable in stratifying individuals for enhancing design of clinical trials, and, hopefully in the near future, for decisions about the risk/benefit of therapeutic interventions, as well.

## Author Contributions

AL reviewed the literature, wrote and edited the manuscript, and made the illustration. BH wrote and edited the manuscript. Both authors contributed to the article and approved the submitted version.

## Conflict of Interest

The authors declare that the research was conducted in the absence of any commercial or financial relationships that could be construed as a potential conflict of interest.
